# Differential Expression of Soluble Receptor for Advanced Glycation End-products in Mice Susceptible or Resistant to Chronic Colitis

**DOI:** 10.1093/ibd/izz311

**Published:** 2019-12-16

**Authors:** Michael Bramhall, Kevin Rich, Ajanta Chakraborty, Larisa Logunova, Namshik Han, James Wilson, John McLaughlin, Andy Brass, Sheena M Cruickshank

**Affiliations:** 1 Department of Biochemistry and Molecular Biology, School of Biomedical Sciences, Monash University, Clayton, Victoria, Australia; 2 Lydia Becker Institute of Immunology and Inflammation, Manchester Academic Health Science Centre, School of Biological Sciences, Faculty of Biology, Medicine, and Health, University of Manchester, Manchester, UK; 3 Manchester Academic Health Science Centre, School of Medical Sciences, The University of Manchester, Manchester, UK; 4 Milner Therapeutics Institute, University of Cambridge, Cambridge, UK; 5 Epistem Ltd., Manchester, UK; 6 Salford Royal NHS Foundation Trust, Salford, UK; 7 Division of Diabetes, Endocrinology and Gastroenterology, School of Medical Sciences, Faculty of Biology, Medicine, and Health, University of Manchester, Manchester, UK

**Keywords:** sRAGE, colitis, mouse

## Abstract

**Background:**

Identifying the factors that contribute to chronicity in inflamed colitic tissue is not trivial. However, in mouse models of colitis, we can investigate at preclinical timepoints. We sought to validate murine *Trichuris muris* infection as a model for identification of factors that promote development of chronic colitis.

**Methods:**

We compared preclinical changes in mice with a resolving immune response to *T. muris* (resistant) vs mice that fail to expel the worms and develop chronic colitis (susceptible). Findings were then validated in healthy controls and patients with suspected or confirmed IBD.

**Results:**

The receptor for advanced glycation end products (RAGE) was highly dysregulated between resistant and susceptible mice before the onset of any pathological signs. Increased soluble RAGE (sRAGE) in the serum and feces of resistant mice correlated with reduced colitis scores. Mouse model findings were validated in a preliminary clinical study: fecal sRAGE was differentially expressed in patients with active IBD compared with IBD in remission, patients with IBD excluded, or healthy controls.

**Conclusions:**

Preclinical changes in mouse models can identify early pathways in the development of chronic inflammation that human studies cannot. We identified the decoy receptor sRAGE as a potential mechanism for protection against chronic inflammation in colitis in mice and humans. We propose that the RAGE pathway is clinically relevant in the onset of chronic colitis and that further study of sRAGE in IBD may provide a novel diagnostic and therapeutic target.

## INTRODUCTION

Inflammatory bowel diseases (IBDs) are intestinal immune disorders, including Crohn’s disease (CD) and ulcerative colitis (UC), that cause chronic inflammation in the gut.^1^ The causes of IBD are currently unknown, but dysregulation of intestinal immunity, microbial dysbiosis, genetics, and environmental factors contribute to disease onset. Unpredictable cycles of remission and relapse require careful monitoring, and long-term damage from inflammation often warrants immunomodulatory therapy or surgical intervention.^2^

Onset, relapse, and remission of IBD are unpredictable, and currently only animal models provide a means of studying the perturbations in the gut that precede colitis. Infecting susceptible mouse strains with the enteric nematode parasite *Trichuris muris* closely parallels human Crohn’s disease both pathologically and transcriptionally.^3–5^*Trichuris muris* resistant BALB/c and C57BL6 mice mount an early dendritic cell (DC) response against the worms within 24 hours of infection, whereas AKR mice or low-dose infected C57BL/6 mice mount a delayed immune response, fail to expel the worms, and develop chronic intestinal inflammation.^5, 6^ Both susceptible and resistant strains show mild signs of inflammation within 24 hours. However, inflammation in resistant mice is controlled and resolves, whereas susceptible strains develop clinical colitis in the weeks after infection.

Very early host immune responses to *T. muris* infection may dictate the onset of chronic, rather than resolving, inflammation in the gut.^7^ However, these factors are impossible to distinguish from the inflammatory milieu present in IBD patients, as chronic inflammation is already established. Identification of early changes during colitis onset in mice could provide relevant IBD biomarkers for human colitis.

We carried out a *T. muris* infection study investigating preclinical transcriptional changes 24 hours postinfection (PI). The receptor for advanced glycation end-products (RAGE) was highly upregulated in mice susceptible to *T. muris* infection. We further investigated the presence of RAGE and related ligands in colitic mice and carried out a translational validation study investigating the presence of soluble RAGE (sRAGE) in the feces of IBD patients and healthy controls.

## MATERIALS AND METHODS

### Mice

Six to eight-week old male BALB/c and AKR mice (Harlan UK, Bicester, UK) were housed in individually ventilated cages with nesting material and maintained under a constant 12-hour light-dark cycle at 21 to 23°C with free access to water and standard chow (Beekay Rat and Mouse Diet, Bantin & Kingham, Hull, UK). Euthanasia was carried out by schedule 1 procedure of CO_2_ asphyxiation followed by cervical dislocation or exsanguination. Three to six mice were used per strain per time point studied.

### Parasites and Infection

Professor Kathryn Else, The University of Manchester, kindly provided eggs of *T. muris* Edinburgh (E) isolate. Egg infectivity and maintenance of parasite stocks were carried out as described by Wakelin.^8^ Experimental mice were infected with 200 embryonated eggs in 200 μL of ultra-pure distilled water via oral gavage. Worm burden was assessed at day 21 PI. Cecum and proximal colon were harvested at autopsy to determine parasite clearance of each mouse at the end of each experiment as described by Else et al.^9^

### Human Samples

Fecal samples were taken from healthy controls (n = 10) with no prior history of IBD or gut problems or patients (n = 31) with suspected IBD or clinically confirmed IBD. All patient samples were taken via outpatient clinics, returned by patients as part of standard clinical practice to be assessed for fecal calprotectin (FCP). Colonoscopy/biopsy were undertaken in those with elevated FCP. Of the patients, 6 patients had IBD excluded, mainly leading to a clinical diagnosis of irritable bowel syndrome (IBS), 19 known IBD patients were in remission at the of time testing (10 ulcerative colitis and 9 Crohn’s disease), and 6 patients had active IBD (n = 5 CD, n = 1 UC) at the time of testing.

### Statistics and Analysis

Experimental groups were compared using linear regression, Mann-Whitney *U* test or 2-way analysis of variance (ANOVA) test followed by the Sidak post hoc multiple comparisons test, where appropriate. *P* values <0.05 were considered significant. Data are presented as mean ± SEM unless otherwise stated. Statistical analyses were carried out using GraphPad Prism 7 (GraphPad Software, La Jolla, California, USA; www.graphpad.com).

### Ethical Considerations

All animal procedures used in this project were carried out in accordance with the UK Animals (Scientific Procedures) Act of 1986. Before the commencement of the clinical study, National Health Service (NHS) ethics approval was obtained from Berkshire B Research Ethics Committee (REC reference number: 14/SC/1413; IRAS reference number: 157778) to screen clinical IBD samples of feces. Participants were recruited from the Salford Royal NHS Foundation Trust. Normal healthy volunteers were recruited in accordance with the university ethics committee and the Human Tissue Act of 2004.

## RESULTS

### Early Immune Response Informs Resistance to *Trichuris Muris*–induced Colitis

**FIGURE 1.**
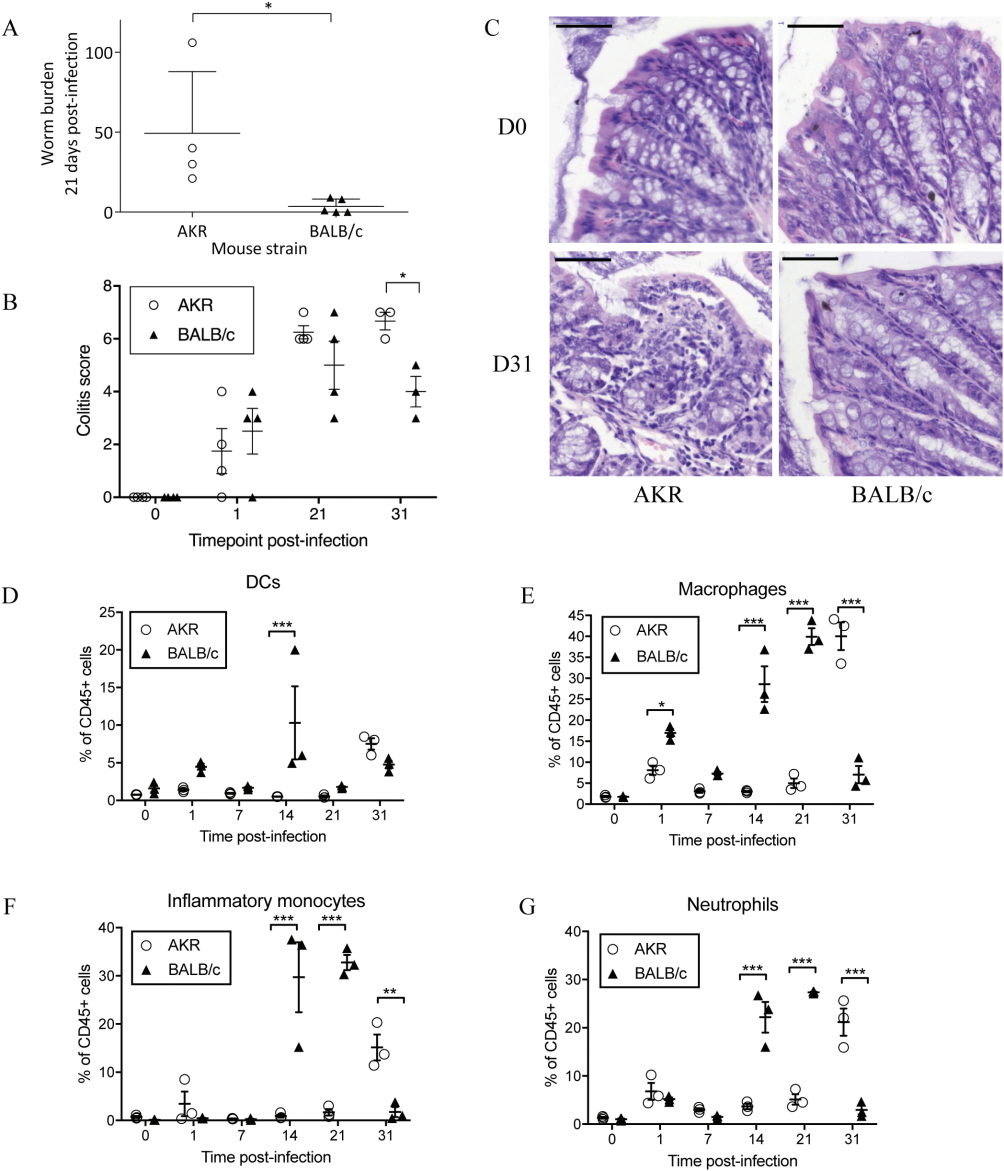
Colitis-susceptible AKR mice show delayed expulsion of *Trichuris muris* worms at 21 days and increased evidence of colitis at 31 days postinfection. (A) Mean worm burden (±SD) at 21 days postinfection. (B) Cumulative colitis score (0–20) based on the grading of histological changes including crypt elongation (score 0–4), depletion of goblet cells (score 0–4), thickness of muscle wall (score 0–4), inflammatory cell infiltration (score 0–4), and destruction of architecture (score 0 or 3–4). (C) Representative images of hematoxylin and eosin stained proximal colon sections from naïve mice and at 31 days PI; note the high levels of immune cell infiltration and loss of goblet cells in the colonic tissues of AKR mice at 31 days postinfection. Bar = 50 μm, n = 3–5 mice per time point. (D–G) Dendritic cell (CD45^+^ MHCII^+^ CD11c^+^ F4/80^-^ CD103^+/-^ CD11b^+/-^), macrophage (CD45^+^ MHCII^+^ F4/80^+^ CD11c^+/-^), inflammatory monocyte (CD45^+^ Ly6G^+^ CD11b^+^ CD115^+^), and neutrophil (CD45^+^ Ly6G^+^ CD11b^+^ CD115^-^) populations as proportion of CD45^+^ cells (±SEM) in naïve mice and during *T. muris* challenge; n = 3 mice per time point. Analysis by Mann-Whitney *U* test or 2-way ANOVA with the Sidak multiple comparisons post hoc test. ***P < 0.001, **P < 0.01, *P < 0.05.

As expected, BALB/c mice expelled most or all of the worms by 21 days PI, whereas AKR mice were unable to expel all worms and remained infected with a significantly higher worm burden (*P* = 0.016, Mann-Whitney *U* test) (Fig. 1A). Colitis scoring revealed inflammation in both AKR and BALB/c mice after infection (Fig. 1B). Changes included influx of immune cells, presence of immune cells in the submucosa, crypt hyperplasia, and goblet cell loss. In agreement with previous data, the colitis scores in BALB/c mice peaked at 21 days PI and began returning to normal by 31 days PI. Colitis scores in AKR mice rose after infection and peaked at 31 days PI when the colitis score was significantly greater than BALB/c mice (*P* = 0.046, ANOVA; Fig. 1B). Hematoxylin and eosin (H&E) stained proximal colon sections are shown in Figure 1C. Collectively, these results reproduce previously published AKR/BALB/c infection data, in which BALB/c mice initiate acute, resolving inflammation after *T. muris* challenge and AKR mice display chronic inflammation due to a failure to expel worms.^4^

We investigated immune cell recruitment into the colonic lamina propria by flow cytometry. The BALB/c mice responded rapidly to *T. muris* challenge, with increased DCs, macrophages, and neutrophils (*P* < 0.05, ANOVA) in colonic lamina propria and mesenteric lymph nodes compared with naïve mice at day 1 PI (Fig. 1D and 1E, Supplementary data). The BALB/c and AKR mice had different responses to infection; BALB/c mice recruited more innate immune cells between day 1 and day 14 compared with susceptible AKR mice, with the greatest difference at day 14 (Fig. 1B; see online supplementary data). By day 31, proportions of macrophages (*P* < 0.001), inflammatory monocytes (*P* < 0.01), and neutrophils (*P* < 0.001) were all significantly greater in AKR mice (Fig. 1D–G). Increased immune cells observed in AKR mice at day 31 corresponded with peak colitis (Fig. 1B). Based on these observations, we then explored early transcriptional responses to *T. muris* to identify intrinsic differences between colitis-susceptible and colitis-resistant mice.

### Transcriptional Changes Induced by *Trichuris Muris* Infection Identified at 24 Hours Postinfection

**FIGURE 2.**
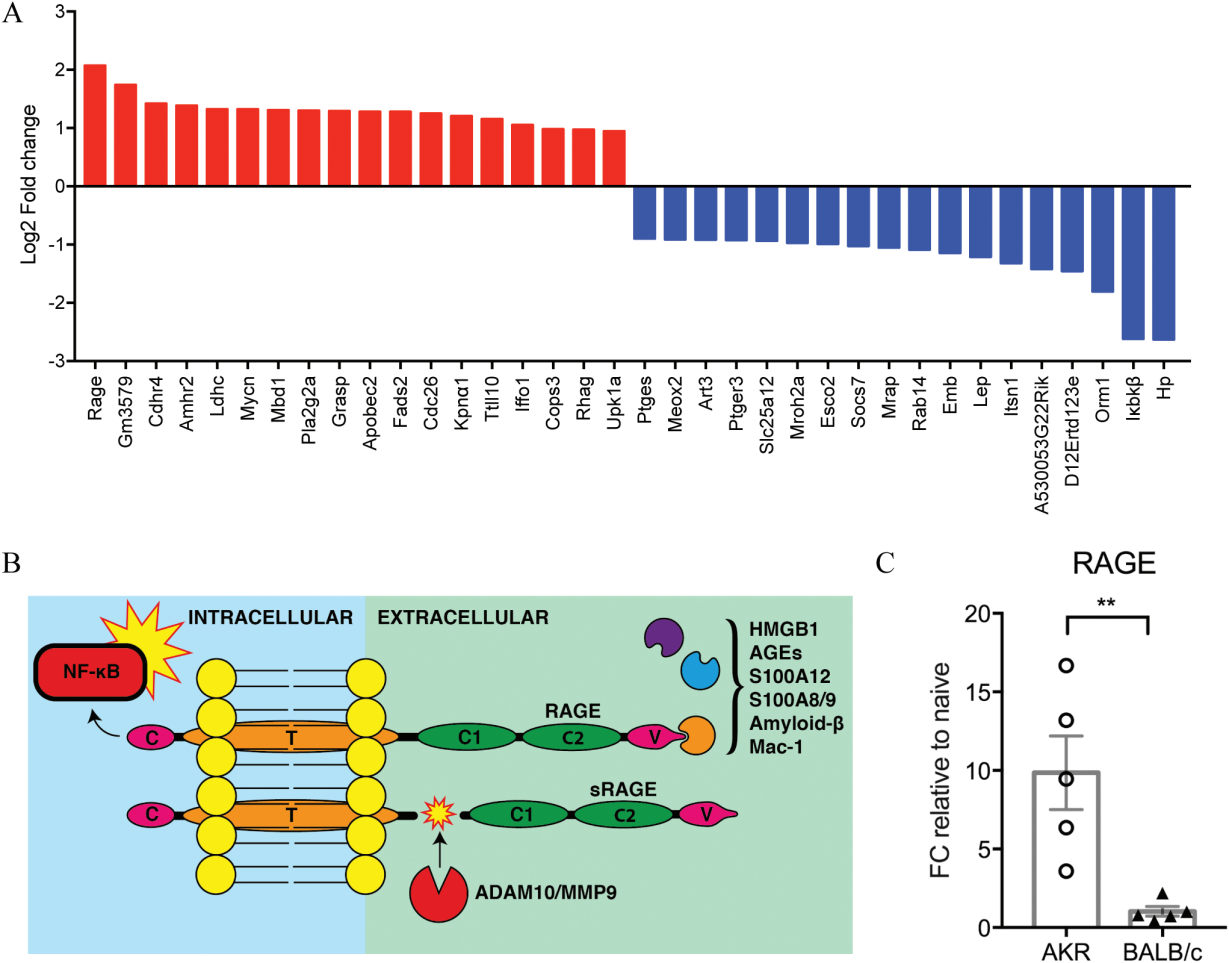
Gene expression changes in proximal colon 24 hours postinfection with *Trichuris* muris. (A) Genes most significantly upregulated in AKR mice and downregulated in BALB/c mice (red) or downregulated in AKR mice and upregulated in BALB/c mice (blue) following 24 hour *Trichuris muris* infection. (B) Schematic of the structure of RAGE, showing activating ligands, downstream NF-κB activation, and formation of soluble RAGE (sRAGE) by ADAM10 or MMP9 cleavage. (C) mRNA expression of RAGE in the proximal colon at day 1 postinfection as measured by qPCR. Data generated using Affymetrix Mouse 430 2.0 microarrays analyzed using the puma and TIGERi (TFA illustrator for global explanation of regulatory interactions) packages for Bioconductor; n = 4–5 mice per group. Analysis by Mann-Whitney *U* test. **P < 0.01.

Transcriptional changes in the proximal colon (the principal site of *T. muris* infection) were investigated at 24 hours postinfection via microarray before the establishment of overt signs of inflammation. Genes with the largest differential expression at 24 hours postinfection were calculated (Fig. 2A). Seventy-seven probe sets were significantly upregulated in AKR mice and downregulated in BALB/c mice (1-IPPLR < 0.05), 65 of which were matched to gene IDs in DAVID. The most differentially expressed upregulated gene in AKR mice compared with BALB/c mice was the receptor for advanced glycation end-products (RAGE) (Log2 fold change = 2.0718; 1-IPPLR = 0.003) (Fig. 2B; see online supplementary data). Upregulation of RAGE in susceptible mice was confirmed by quantitative polymerase chain reaction (qPCR) of proximal colon at 24 hours PI in an independent experiment (*P* < 0.001, Mann-Whitney *U* test, Fig. 2C).

Differential expression of transcription factors was analyzed using TIGERi for MATLAB.^10^ Forkhead box O4 (FOXO4) was notably downregulated in AKR mice (see online supplementary data). Forkhead box O4 occurs downstream of RAGE signalling and serves to inhibit DNA binding and transcriptional activity of NF-κB (nuclear factor kappa-B), preventing inflammation.^11^ Forkhead box O4 is also downregulated in colonic epithelial cells of IBD patients.^11^ Upregulation of pro-inflammatory RAGE and downregulation of anti-inflammatory FOXO4 from the RAGE signalling pathway provides compelling evidence for the relevance of RAGE activation in colitis susceptibility during *T. muris* infection.

### Identifying the Cellular Source of RAGE

**FIGURE 3.**
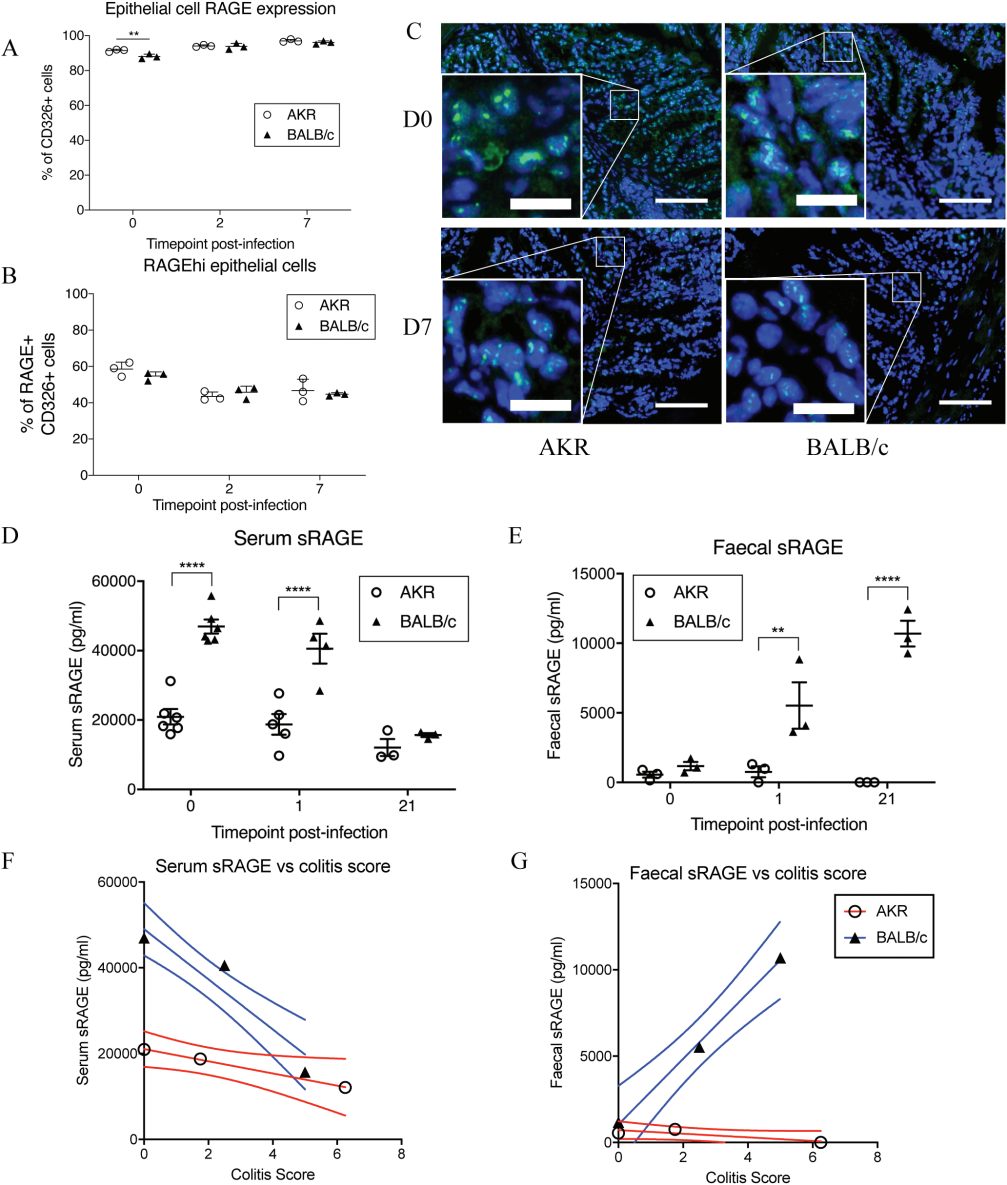
Receptor for advanced glycation end-products expression in the colon of *Trichuris muris* infected AKR and BALB/c mice (aged 6–8 weeks). (A) Proportion of RAGE expressing epithelial cells (CD326^+^, ±SEM) during early *Trichuris muris* infection. (B) Proportion of colonic epithelial cells expressing high levels of RAGE is reduced shortly after infection, as measured by flow cytometry. (C) Representative images of colon sections stained for RAGE (FITC; green) and nuclei (DAPI; blue) in naïve mice and at 7 days postinfection (Bar = 100μm; inset bar = 22μm). (D–E) sRAGE present in serum or feces during *Trichuris muris* infection as measured by ELISA. (F–G) Correlation of serum and fecal sRAGE versus colitis score at 0, 1, and 21 days postinfection; n = 3–5 mice per time point. Analysis by linear regression, 2-way ANOVA with the Sidak post hoc test. **P < 0.01 ****P < 0.001.

Over 90% of CD326^+^ (EpCAM) epithelial cells expressed RAGE (Fig. 3A), and they fell into 2 distinct groups, expressing either low (RAGE^lo^) or high (RAGE^hi^) levels of RAGE. In naïve mice, the total proportion of CD326^+^ epithelial cells that expressed RAGE was significantly higher in colitis-susceptible AKR mice (*P* < 0.01, ANOVA). The proportion of RAGE^lo^ to RAGE^hi^ cells was similar in both naïve AKR and BALB/c mice, but there was a significant drop in the proportion of RAGE^hi^ epithelial cells observed 2 and 7 days PI (*P* < 0.0001, ANOVA) in both AKR and BALB/c mice (Fig. 3B).

Immunohistochemistry was used to confirm expression of RAGE throughout the colonic epithelium (Fig. 3C). Concurring with the flow cytometry data, there was only minimal fluorescence seen in the immune lamina propria cells indicating that epithelial cells indeed express greater amounts of membrane-bound RAGE per cell than immune cells. These data suggest that the epithelial cells are likely to be the source of the observed increase in RAGE mRNA. There was a reduction in intensity of RAGE staining at 7 days PI by immunohistochemistry (Fig. 3C) compared with naïve mice, which also correlated with the measured shift in proportions of epithelial cells from RAGE^hi^ to RAGE^lo^ cells measured by flow cytometry.

### Is RAGE Differentially Cleaved in Colitis-susceptible and colitis-resistant Mice?

The receptor for advanced glycation end-products may be internalized after ligand binding or released as soluble RAGE (sRAGE) via enzymatic cleavage by A disintegrin and metalloproteinase domain-containing protein 10 (ADAM10) or matrix metalloproteinase 9 (MMP9).^12, 13^ To investigate whether RAGE was being cleaved, we assessed sRAGE levels in serum and the feces by ELISA. Serum sRAGE levels in susceptible mice remained constant throughout the experiment. Resistant mice had significantly higher serum sRAGE than susceptible mice before infection and at 24 hours PI (*P* < 0.001, ANOVA; Fig. 3D). Soluble RAGE was also detectable in feces; both resistant and susceptible mice had low levels of sRAGE before infection. Fecal sRAGE in BALB/c mice increased to significantly higher levels than susceptible mice at 24 hours and 21 days PI (*P* < 0.01, ANOVA; Fig. 3E). Fecal sRAGE was not detected at all up to day 21 in susceptible mice.

The correlation of serum and fecal sRAGE levels to colitis scores highlights the changing levels of sRAGE in BALB/c mice during the course of *T. muris* infection relative to pathological changes in the colon (Fig. 3F, G). The increased circulating serum sRAGE at day 0 in BALB/c mice, where colitis scores are lowest, drops as colitis increases at day 1 and day 21 (R^2^ = 0.90). Fecal sRAGE in the BALB/c mice increases relative to colitis scores (R^2^ = 0.99). However, sRAGE levels in susceptible mice did not change during the course of infection relative to increasing colitis scores from day 0 to day 21 postinfection (serum R^2^ = 0.99, fecal R^2^ = 0.73).

We then investigated levels of the RAGE ligand S100A8 (one part of the heterodimeric calprotectin protein, currently used as a clinical biomarker for IBD) in serum and feces as an indicator of whether sRAGE was quenching the effects of circulating RAGE ligands by acting as a decoy receptor. Serum and fecal S100A8 did increase during the course of infection in both AKR and BALB/c mice, but no statistical differences were observed, and there was high variability between mice. At 21 days PI, BALB/c mice had greater levels of serum S100A8 than AKR mice (not significant, ANOVA; see online supplementary data). Fecal S100A8 remained similar in naïve and infected mice of both AKR and BALB/c strains. As with serum S100A8, fecal S100A8 was raized in BALB/c mice at 21 days PI compared with AKR mice, but this was not significant (ANOVA; see online supplementary data). In addition to being highly variable, S100A8 correlated poorly with colitis scores (see online supplementary data).

### sRAGE Is Differentially Expressed in IBD

**FIGURE 4.**
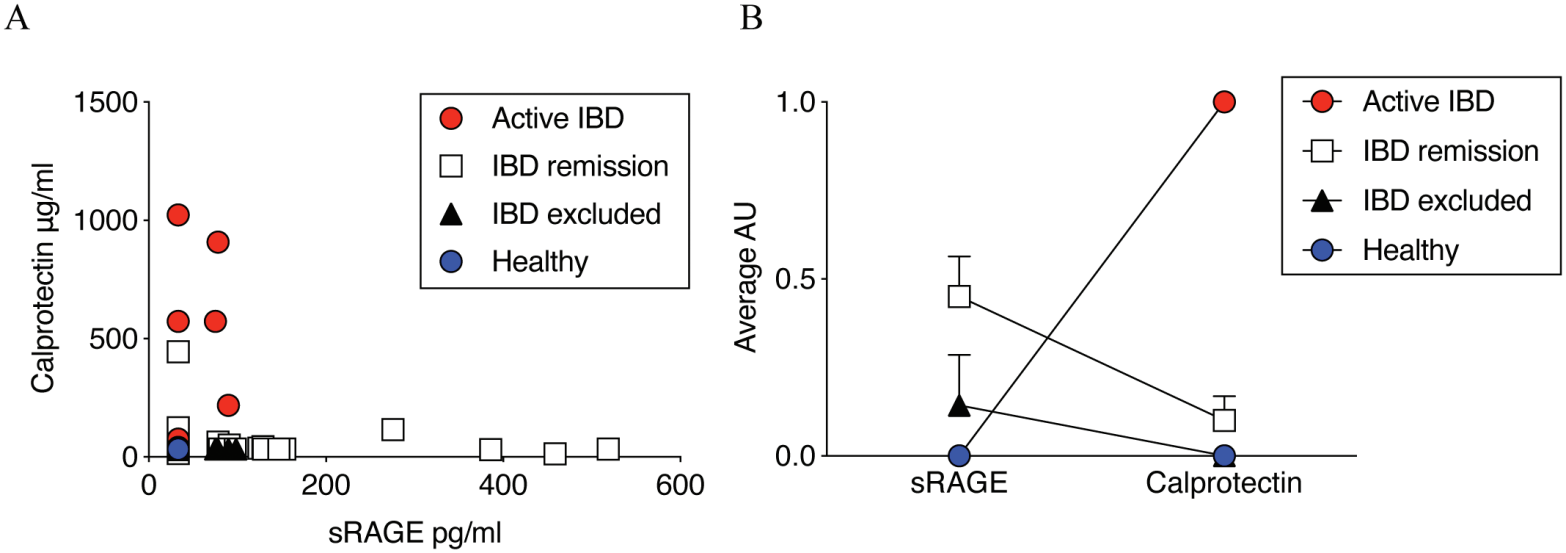
Soluble receptor for advanced glycation end-products is detectable in the feces of IBD patients. (A) Scatter plot of sRAGE (pg/mL) versus calprotectin (μg/mL) present in the feces of patients with active IBD, IBD in remission, and IBD excluded (IBS) compared with healthy controls. (B) Relative levels of fecal sRAGE vs calprotectin in human IBD/IBS or healthy controls. Data were transformed to arbitrary units (AU) where samples greater than 3 SD from baseline = 1 (present); otherwise they scored 0 (absent).

As the differences in sRAGE were most apparent and consistent in feces, we focused on analysis of fecal specimens from healthy volunteers and patients with IBD or suspected IBD. Soluble RAGE was not detected in the fecal samples of healthy volunteers but was detectable in the patient cohort (Fig. 4A). The highest levels of sRAGE were seen in patients with IBD excluded (largely IBS ascribed) and IBD in remission, although remission patients were more variable. Patients with active IBD characterized by severe inflammation had low levels of sRAGE and increased calprotectin. We then transformed the fecal RAGE and calprotectin ELISA data into present (1) or absent (0), where protein is scored as present if ≥3 SD above baseline. When patient data were stratified into groups (active IBD, IBD in remission, IBD excluded [IBS] and healthy controls) the ratio of RAGE to calprotectin clearly identified healthy controls (0, 0) and active IBD (0, 1) from IBS and IBD in remission (Fig. 4B). In healthy individuals, we saw no sRAGE or calprotectin signal in any subject. In active IBD, we had a consistent pattern of calprotectin present but no sRAGE. For patients whose disease was resolving, we saw a more complicated picture in which we had a subset of patients undergoing routine tests that were expressing both sRAGE and calprotectin.

## DISCUSSION

Genetic factors influence the likelihood of developing colitis, but continually monitoring susceptible individuals is unfeasible. Animal models remain crucial for examining the causative events that lead to chronic diseases. Levison et al have previously shown that *T. muris* infection in AKR mice causes colitis that correlates phenotypically and transcriptionally with the profile of human CD.^14^ Here we provide novel evidence for the *T. muris* infection as a model for the discovery of preclinical intestinal inflammation markers that translate into human IBD patients.

In line with previous *T. muris* infection studies, we observed an early influx of DCs in colitis-resistant mice.^5^ We now identify upregulation of RAGE as an indicator of colitis susceptibility in mice. Activity of RAGE has already been linked to active IBD, diabetes, Alzheimer’s, airway inflammation, cancer, and hemorrhagic shock.^15, 16^ Additionally, several RAGE ligands are associated with inflammation in IBD, including calprotectin, EN-RAGE, and high mobility group box 1 (HMGB1).^17–19^ It is important to validate results from mouse model to human disease, and therefore, we conducted a small validation study to assess fecal sRAGE in human patients. Fecal sRAGE was readily detected in patient fecal samples. Akin to the mouse data, we saw lower sRAGE in patients with active chronic inflammation. Surprisingly, symptomatic patients with IBD excluded on a basis of normal FCP and/or colonoscopy had higher levels of sRAGE than healthy volunteers. Largely, IBS was clinically ascribed, but that was not based on formal diagnostic criteria, and other diagnoses such as bile acid diarrhea or microscopic colitis were not formally excluded. The sample size was small, and more prospective studies are needed to confirm this preliminary observation. Patients whose IBD was reported to be in remission had variable levels of sRAGE. It is tempting to speculate that lower levels of sRAGE are associated with a risk of subsequent flare of inflammation, but because we only had single samples from the patients, we cannot assess this; however, it would be interesting to track sRAGE over time in a prospective study of patients with IBD. Additionally, further analysis of the levels of circulating sRAGE in patient serum would also provide useful comparisons among healthy patients, IBD excluded patients, and patients with IBD or IBD in remission, but we were unable to obtain sufficient serum samples in this study. Analysis of fecal and serum sRAGE in other chronic inflammatory diseases may also provide useful information to determine whether levels of RAGE and sRAGE are more general markers of chronic inflammation and resolution, respectively. Several studies suggest that circulating sRAGE is protective against various inflammatory diseases including atherosclerosis, coronary artery disease, hypertension, rheumatoid arthritis, and Alzheimer’s disease.^20–22^

The receptor for advanced glycation end-products is expressed by several immune cell types, with neutrophils identified as expressing large amounts of RAGE.^23^ Our flow cytometry and immunohistochemistry analysis of RAGE expression in multiple cell types present in and around the lamina propria and crypts of the colon, however, did not suggest that immune cells were the main sources of cellular RAGE. Our data, in fact, showed that a major cellular source of RAGE in the gut was epithelial cells. Previous studies have shown that epithelial cells not only express RAGE but also upregulate RAGE expression during colonic inflammation.^15^ Changes we observed in the levels of RAGE expression suggest that it is the epithelial cell response to *T. muris* infection that informs subsequent susceptibility to chronic inflammation. The reduction in the amount of RAGE present at the cell membrane we saw by flow cytometry and by immunohistochemistry immediately after *T. muris* infection could be caused by either internalization of activated RAGE-ligand complexes or ADAM10-mediated shedding to produce sRAGE.^24^ Splice variants of RAGE may also result in a truncated RAGE molecule or a modified and actively secreted decoy receptor.^25^ The process by which epithelial cells may undergo RAGE shedding represents an important distinction in the course of gut immunity and homeostasis and may dictate whether inflammation becomes chronic or resolves. Further investigation into splice variants, internalization, or sheddase activity could provide further insight into the mechanism by which the RAGE pathway influences colitis.

The receptor for advanced glycation end-products mediates homing of DCs to the lymph nodes via CD11b (Mac-1).^15^ However, whether the upregulation of RAGE is crucial to facilitate the early DC migration in the *T. muris* model remains unclear. Although we observed differences in DC migration as early as day 1 PI, we saw no differences between cell surface RAGE expression between AKR and BALB/c mice that might account for the altered dynamics of DC recruitment. Similarly, RAGE is linked to neutrophil recruitment, but we only observed modest neutrophil infiltration at 24 hours PI and no difference between colitis-susceptible or colitis-resistant mice. Despite differences in the presence of immune cells during early *T. muris* infection, prolonged activation of RAGE results in activation of inflammatory signalling molecules, including NF-κB and MAP kinases.^16^ Consequently, an environment where RAGE ligands such as HMGB1 and S100 proteins are continually present results in perpetual NF-κB activation and subsequent chronic inflammatory conditions.

We observed striking differences in the levels of fecal and systemic sRAGE between colitis-resistant and colitis-susceptible mice, with BALB/c mice rapidly producing sRAGE in response to *T. muris* infection. Soluble RAGE acts as a decoy receptor for RAGE, binding to the same damage-induced ligands as membrane-bound RAGE, but because it lacks a cytoplasmic tail, it cannot initiate pro-inflammatory signalling.^26^ Thus, higher levels of sRAGE might block inflammation, and reduced levels of sRAGE have been found in mice with chronic inflammation and in patients with chronic inflammatory diseases.^22^ Reduced epithelial RAGE expression followed by increased sRAGE in colitis-resistant mice suggests shedding of RAGE as a protective response against the development of chronic inflammation. Colitis-susceptible AKR mice showed the same reduction in epithelial RAGE expression but did not produce sRAGE in the same quantities, suggesting internalization and pro-inflammatory activation of RAGE after *T. muris* infection. Indeed, it is known that *T. muris* excretory/secretory (E/S) products induce NF-κB signalling in colonic epithelial cells shortly after infection, and the susceptible immune response to *T. muris* is associated with expression of the T helper (T_H_) 1 cytokines interferon (IFN)-γ, tumur necrosis factor (TNF)-α, and interleukin (IL)-12.^27^ This response parallels the cytokines expressed after RAGE activation, which include T_H_1 and T_H_17 cytokines TNF-α, IL-1α, IL-6, IL-8, and IL-12.^16^

Diagnosis of IBD involves an assessment of clinical history and physical examination, with endoscopy and histology considered to be the gold standard tools.^28^ Accurately assessing disease activity remains dependent on colonoscopy and/or small bowel imaging. Current diagnostic methods for assessment of IBD are invasive and thus there is a need to identify serum of fecal biomarkers that can reliably monitor IBD disease.^29^ The number of potential IBD biomarkers is high, but reliable and reproducible biomarkers in clinical practice are scarce.^30^ Response to current therapies is also variable, and there is considerable interest for new biomarkers and new therapeutics.^31^ Calprotectin is a noninvasive aid to clinical diagnosis, but measurements of fecal calprotectin are variable; and the normal baseline level for healthy patients is under debate.^32^ Indeed, the concept of a simple normal cutoff is difficult to entertain given the enormous heterogeneity in fecal water content, matrix composition, transit time, site and extent of inflammation, and the contact of fecal component sampled with the mucosa; composite measures are essential. Calprotectin is a product of tissue damage and binds to RAGE to promote inflammation, but this action will be reduced in the presence of the decoy receptor sRAGE. This is also reflected in the observation that overexpression of RAGE and reduced sRAGE correlates with chronic inflammation in inflammatory diseases such as atherosclerosis and diabetes.^22^ Our preliminary observation of alterations in sRAGE expression in mouse and human disease suggests there may be merit in looking at both calprotectin and sRAGE to better predict whether calprotectin and other RAGE ligands are indeed able to drive pro-inflammatory signals. This may improve the reliability of calprotectin as a biomarker. Further studies investigating the ratios and functions of RAGE and sRAGE and the mechanisms by which RAGE is cleaved or secreted by cells in other systemic inflammatory diseases would also provide useful insights into the onset and resolution of chronic inflammation. Additional comparison to an alternative inflammatory disease to control for inflammatory factors from causes other than IBD, such as rheumatoid arthritis, would also be interesting to assess whether such factors are specific to IBD or more general markers of inflammation.

## CONCLUSION

Our pilot clinical study successfully validated the use of the *T. muris* infection model as highly translatable to human IBD states. The *T. muris* model is a useful tool in dissecting early pathways that are involved in the onset of colitis. By focusing on early initiating events in the development of colitis, we have identified a potential role for RAGE in mediating the development of inflammation. Furthermore, our observation of high levels of sRAGE in acute resolving inflammation suggested there may be utility in monitoring sRAGE to monitor IBD. However, given the small size and limitations of this study, a larger clinical study with additional control samples from another chronic inflammatory disease cohort would be required to investigate more fully the possibility of combining fecal sRAGE and calprotectin levels as a reliable biomarker for resolution of colitis.

## Supplementary Material

izz311_suppl_Supplementary_Data

## Notes

Supported by: Funding was provided by an EPSRC case studentship awarded to MB sponsored by Engineering and Physical Sciences Research Council, Biotechnology and Biological Sciences Research Council, Epistem Ltd. and the BBSRC impact accelerator fund awarded to SC, AB, and JM.

## References

[1] Baumgart DC, Carding SR Inflammatory bowel disease: cause and immunobiology. Lancet. 2007;369:1627–1640. 17499605 10.1016/S0140-6736(07)60750-8

[2] Kaplan GG The global burden of IBD: from 2015 to 2025. Nat Rev Gastroenterol Hepatol. 2015;12:720–727. 26323879 10.1038/nrgastro.2015.150

[3] Liverani E, Scaioli E, Digby RJ, et al. How to predict clinical relapse in inflammatory bowel disease patients. World J Gastroenterol. 2016;22:1017–1033. 26811644 10.3748/wjg.v22.i3.1017 PMC4716017

[4] Levison SE, Fisher P, Hankinson J, et al. Genetic analysis of the *Trichuris muris*-induced model of colitis reveals QTL overlap and a novel gene cluster for establishing colonic inflammation. BMC Genomics. 2013;14:127. 23442222 10.1186/1471-2164-14-127 PMC3621453

[5] Cruickshank SM, Deschoolmeester ML, Svensson M, et al. Rapid dendritic cell mobilization to the large intestinal epithelium is associated with resistance to *Trichuris muris* infection. J Immunol. 2009;182:3055–3062. 19234202 10.4049/jimmunol.0802749 PMC2671799

[6] Bowcutt R, Bramhall M, Logunova L, et al. A role for the pattern recognition receptor Nod2 in promoting recruitment of CD103+ dendritic cells to the colon in response to *Trichuris muris* infection. Mucosal Immunol. 2014;7:1094–1105. 24448097 10.1038/mi.2013.125 PMC4074062

[7] Foell D, Wittkowski H, Roth J Monitoring disease activity by stool analyses: from occult blood to molecular markers of intestinal inflammation and damage. Gut. 2009;58:859–868. 19136508 10.1136/gut.2008.170019

[8] Wakelin D Acquired immunity to *Trichuris muris* in the albino laboratory mouse. Parasitology. 1967;57:515–524. 6048569 10.1017/s0031182000072395

[9] Else KJ, Wakelin D, Wassom DL, et al The influence of genes mapping within the major histocompatibility complex on resistance to *Trichuris muris* infections in mice. Parasitology. 1990;101 Pt 1:61–67. 2235076 10.1017/s0031182000079762

[10] Han N, Noyes HA, Brass A TIGERi: modeling and visualizing the responses to perturbation of a transcription factor network. BMC Bioinformatics. 2017;18:260. 28617232 10.1186/s12859-017-1636-6 PMC5471961

[11] Zhou W, Cao Q, Peng Y, et al. FoxO4 inhibits NF-kappaB and protects mice against colonic injury and inflammation. Gastroenterology. 2009;137:1403–1414. 19560465 10.1053/j.gastro.2009.06.049 PMC2764529

[12] Curran CS, Bertics PJ Human eosinophils express RAGE, produce RAGE ligands, exhibit PKC-delta phosphorylation and enhanced viability in response to the RAGE ligand, S100B. Int Immunol. 2011;23:713–728. 22025532 10.1093/intimm/dxr083 PMC3226529

[13] Zhang L, Bukulin M, Kojro E, et al. Receptor for advanced glycation end products is subjected to protein ectodomain shedding by metalloproteinases. J Biol Chem. 2008;283:35507–35516. 18952609 10.1074/jbc.M806948200

[14] Levison SE, McLaughlin JT, Zeef LA, et al. Colonic transcriptional profiling in resistance and susceptibility to trichuriasis: phenotyping a chronic colitis and lessons for iatrogenic helminthosis. Inflamm Bowel Dis. 2010;16:2065–2079. 20687192 10.1002/ibd.21326

[15] Body-Malapel M, Djouina M, Waxin C, et al. The RAGE signaling pathway is involved in intestinal inflammation and represents a promising therapeutic target for inflammatory bowel diseases. Mucosal Immunol. 2019;12:468–478. 30542111 10.1038/s41385-018-0119-z

[16] Sparvero LJ, Asafu-Adjei D, Kang R, et al. RAGE (receptor for advanced glycation endproducts), RAGE ligands, and their role in cancer and inflammation. J Transl Med. 2009;7:17. 19292913 10.1186/1479-5876-7-17 PMC2666642

[17] Whitehead SJ, Ford C, Gama RM, et al. Effect of faecal calprotectin assay variability on the management of inflammatory bowel disease and potential role of faecal S100A12. J Clin Pathol. 2017;70:1049–1056. 28735301 10.1136/jclinpath-2017-204340

[18] Chen X, Li L, Khan MN, et al. HMGB1 exacerbates experimental mouse colitis by enhancing innate lymphoid cells 3 inflammatory responses via promoted IL-23 production. Innate Immun. 2016;22:696–705. 27670944 10.1177/1753425916669862

[19] Aranda CJ, Ocón B, Arredondo-Amador M, et al. Calprotectin protects against experimental colonic inflammation in mice. Br J Pharmacol. 2018;175:3797–3812. 30007036 10.1111/bph.14449 PMC6135788

[20] Detzen L, Cheng B, Chen CY, et al. Soluble forms of the receptor for advanced glycation endproducts (RAGE) in periodontitis. Sci Rep. 2019;9:8170. 31160611 10.1038/s41598-019-44608-2 PMC6547730

[21] Dozio E, Vianello E, Bandera F, et al. Soluble receptor for advanced glycation end products: a protective molecule against intramyocardial lipid accumulation in obese zucker rats? Mediators Inflamm. 2019;2019:2712376. 30944546 10.1155/2019/2712376 PMC6421753

[22] Maillard-Lefebvre H, Boulanger E, Daroux M, et al. Soluble receptor for advanced glycation end products: a new biomarker in diagnosis and prognosis of chronic inflammatory diseases. Rheumatology (Oxford). 2009;48:1190–1196. 19589888 10.1093/rheumatology/kep199

[23] Huebener P, Pradere JP, Hernandez C, et al. The HMGB1/RAGE axis triggers neutrophil-mediated injury amplification following necrosis. J Clin Invest. 2019;130:1802. 10.1172/JCI126976 PMC6436855 30829652

[24] Yang WS, Kim JJ, Lee MJ, et al. Ectodomain shedding of RAGE and TLR4 as a negative feedback regulation in high-mobility group box 1-activated aortic endothelial cells. Cell Physiol Biochem. 2018;51:1632–1644. 30497069 10.1159/000495651

[25] Jules J, Maiguel D, Hudson BI Alternative splicing of the RAGE cytoplasmic domain regulates cell signaling and function. PLoS One. 2013;8:e78267. 24260107 10.1371/journal.pone.0078267 PMC3832623

[26] Basta G, Sironi AM, Lazzerini G, et al. Circulating soluble receptor for advanced glycation end products is inversely associated with glycemic control and S100A12 protein. J Clin Endocrinol Metab. 2006;91:4628–4634. 16926247 10.1210/jc.2005-2559

[27] deSchoolmeester ML, Manku H, Else KJ The innate immune responses of colonic epithelial cells to *Trichuris muris* are similar in mouse strains that develop a type 1 or type 2 adaptive immune response. Infect Immun. 2006;74:6280–6286. 17057095 10.1128/IAI.01609-05 PMC1695505

[28] Halfvarson J Genetic epidemiology of inflammatory bowel disease, early twin and family studies. In: D’amato M, Rioux JD, eds. Molecular Genetics of Inflammatory Bowel Disease. New York: Springer; 2013:23–43.

[29] Lehmann FS, Burri E, Beglinger C The role and utility of faecal markers in inflammatory bowel disease. Therap Adv Gastroenterol. 2015;8:23–36. 10.1177/1756283X14553384 PMC4265086 25553077

[30] Stevens TW, Matheeuwsen M, Lönnkvist MH, et al. Systematic review: predictive biomarkers of therapeutic response in inflammatory bowel disease-personalised medicine in its infancy. Aliment Pharmacol Ther. 2018;48:1213–1231. 30378142 10.1111/apt.15033

[31] Nanau RM, Cohen LE, Neuman MG Risk of infections of biological therapies with accent on inflammatory bowel disease. J Pharm Pharm Sci. 2014;17:485–531. 25579431 10.18433/j3gg6d

[32] Dhaliwal A, Zeino Z, Tomkins C, et al. Utility of faecal calprotectin in inflammatory bowel disease (IBD): what cut-offs should we apply? Frontline Gastroenterol. 2015;6:14–19. 25580205 10.1136/flgastro-2013-100420 PMC4283700

